# Quantifying shortening of the fractured clavicle assuming clavicular symmetry is unreliable

**DOI:** 10.1007/s00402-018-2912-2

**Published:** 2018-03-13

**Authors:** Paul Hoogervorst, Anand Appalsamy, Sebastiaan Franken, Albert van Kampen, Gerjon Hannink

**Affiliations:** 1 Department of Orthopedics, Radboud University Medical Center, P.O. Box 9101, 6500 HB Nijmegen, The Netherlands; 2 Department of Radiology, Radboud University Medical Center, Nijmegen, The Netherlands

**Keywords:** Clavicle, Length, Symmetry, Interobserver agreement, Imaging

## Abstract

**Background:**

One of the more commonly used methods of determining the amount of shortening of the fractured clavicle is by comparing the length of the fractured side to the length of contralateral unfractured clavicle. A pre-existing natural asymmetry can make quantification of shortening using this method unreliable. The goal of this study is to assess the side-to-side variation in clavicle length in 100 uninjured, skeletally mature adults.

**Materials and methods:**

To assess the side-to-side difference in clavicle length the length of both clavicles of 100 patients on thoracic computed tomography (CT) scans were measured. Patients without a history of pre-CT clavicular injury were included. The measurements were allocated into three groups based on the amount of asymmetry (< 5, ≥ 5–10 and > 10 mm). Dominant side and sex were analyzed to determine influence on the length of the clavicle.

**Results:**

In 30 patients (30%), an asymmetry of 5 mm or more was found. 2% of the patients had a side-to-side difference of more than 10 mm. The absolute side-to-side length difference (LD) was 3.74 mm (95% CI 3.15–4.32; *p* < 0.001). A significant association between clavicle length and dominant side or sex was found (*p* < 0.001).

**Conclusion:**

These results show that by utilizing a treatment algorithm based upon clavicular symmetry has a potential for error and can lead over- or under-treatment of the fractured clavicle. A significant association between clavicle length and dominant side or sex was found (*p* < 0.001).

**Level of evidence:**

2.

## Introduction

Clavicle fractures are common fractures with a prevalence of 59.3 per 100,000 person-years [1]. The majority of these fractures are shortened and/or displaced due to the specific anatomy and muscle insertions. There is still no consensus on how to treat these displaced and/or shortened midshaft clavicle fractures (DMCF). Operative treatment leads to better rates of union, less mal-unions, and increased patient satisfaction in comparison to conservative therapy, but it is accompanied by a higher rate of adverse events [2, 3]. A recent meta-analysis by Kong et al. [4] of six randomized-controlled trails (RCTs) comparing conservative and operative treatments supports these findings. Other studies report on increased pain, loss of strength, rapid fatigue, hyperesthesia of the hand and arm, difficulty sleeping on the affected side, and aesthetic complications in conservatively treated, malunited, and shortened clavicles [5–7]. These may be the reasons why in recent years there is a tendency to surgically reduce and fixate DMCF [5–10]. Current treatment paradigms support the indication for surgery if the fractured clavicle is shortened more than 15–20 mm, or displaced more than the diameter of the clavicle’s shaft [2, 5, 7, 9, 11–13].

Since the clavicle has a sigmoid shape in two planes, adequately quantifying shortening of the fracture elements is challenging. Other variables influencing measurements on the fractured clavicle are patient positioning, magnification, and direction of the X-rays [14–16]. Various methods to quantify shortening, such as clinical measurements and the use of CT scans, have been described [16–18].

A commonly used technique is using AP and 15° caudo-cranial views. However, there are papers which support the use of a 15–30° cranio-caudal AP or PA views. In addition, a PA thorax view is used in measuring the shortening of DMCF [14, 16–18]; Silva et al. [17] proposed a standardized method of measuring shortening in DMCF, even though no better interobserver agreement was shown.

It remains unclear which method or technique would be best to quantify shortening of the fractured clavicle.

Another commonly used method is to determine the amount of shortening by comparing the fractured side to the contralateral unfractured clavicle on a panoramic AP view of both clavicles. This presumes clavicular symmetry. To our knowledge, there is only one study that investigated clavicular symmetry. Cunningham et al. [19] reported an asymmetry of clavicular length of 5 mm or more in 28.5% of the studied population and found no association between side-to-side differences and sex. However, they did not exclude pre-CT clavicular injuries or investigate associations between side-to-side differences and hand dominance. This might be valuable, since hand dominance is associated with differences in upper limb bone mineralization and hand size [20, 21].

Because, in recent years, an absolute shortening of 15 mm is thought to be a relative indication for surgery, [5, 9, 13], a pre-existing asymmetry of 5 mm or more in an important part of the population may lead to the conclusion that quantifying shortening using this method is unreliable [19].

The goal of this study was to assess the side-to-side difference in clavicle length in 100 skeletally mature adults without any pre-CT clavicular injuries, and to investigate possible associations between clavicular length and sex or hand dominance.

## Methods

### Design

To assess the side-to-side differences in clavicle length, we measured the length of both clavicles of 100 patients on 100 thoracic computed tomography (CT) scans. The study protocol was approved by our institutional review board (CMO 2014-1432).

### Patients

Each thoracic CT scan that was made between September 2014 and February 2015 in our institution for any reason was first assessed if both clavicles were completely and adequately imaged. If so, we contacted each patient of whom we would like to use the CT scan. During the phone interview, verbal consent was given by patient involved to use their images. All patients were over 18 years old. Only those patients without a history of pre-CT clavicular injury were included. All patients included stated their dominant side.

A total of 132 scans were evaluated. Two patients did not want their thoracic CT scan to be included. Twelve patients could not be reached on multiple occasions. Three patients were deceased. Fifteen were excluded due to a clavicular fracture in the past.

### Measurements

**Fig. 1:**
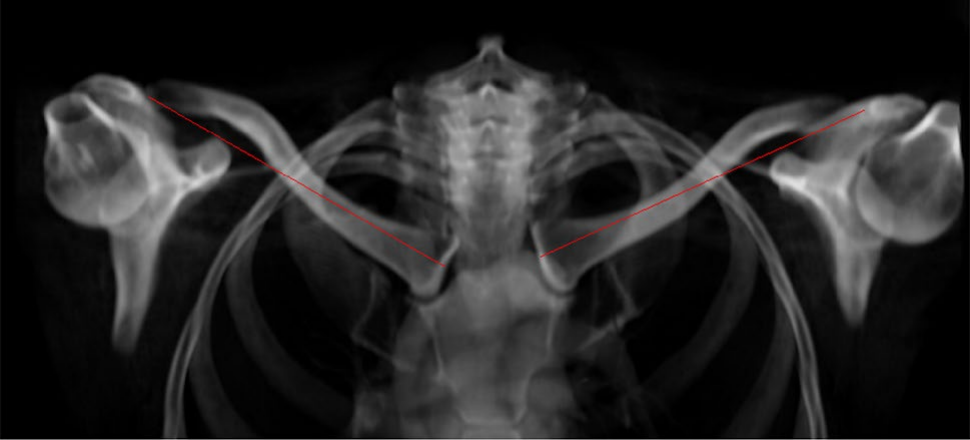
Example of measurements on 3D reconstruction of a CT scan showing both clavicles

Two observers [SF (radiologist) and AA (medical student)] measured both clavicles in random order on a 3D reconstruction of the CT scan using TeraRecon Aquarius Intuiton (Foster City, CA, USA). Measurements on a patient’s right and left clavicle were performed on separate occasions at least 2 weeks apart to prevent bias. Before the start of the study, a training session with both observers took place and the measurement methodology was standardized. The observers agreed upon the precise definitions of the reference points. The reconstructions were projected in such a way that the length of the clavicle was maximized according to the observer. Clavicle length was defined as the distance between the lateral-most point of the clavicle in the acromioclavicular joint and the medial-most point of the clavicle in the sternoclavicular joint. The clavicle length was measured between these points using the same software (Fig. 1). The absolute side-to-side length difference (LD) between the right and left clavicles was calculated by subtracting the length of the short side (SS) from the length of the long side (LS). The LD were categorized into three groups based on the amount of asymmetry. One group included all patients in which the side-to-side difference was < 5 mm. The other two groups consisted of those patients with an asymmetry of ≥ 5–10 mm and > 10 mm side-to-side difference. These criteria were chosen, since a 5 mm side-to-side difference might be clinically relevant when deciding on a surgical intervention of the fractured clavicle.

### Statistical analysis

Interobserver agreement was assessed by calculation of concordance correlation coefficients (CCC). The CCC for repeated measurements (left and right clavicle) were estimated using the variance components from a linear mixed model estimated by restricted maximum likelihood [22, 23]. Limits of agreement (LoA) were calculated to assess systematic and random measurements error between both observers.

Measurements were only performed once, since intraobserver agreement for measurements performed on CT scans is known to be high [19, 24]. Measurements of both observers were averaged when interobserver agreement was almost perfect (i.e., CCC ≥ 0.99) [25].

Descriptive statistics were used to summarize the data. Paired sample *t* tests were used to test side-to-side length differences. The associations between clavicle length and dominant side and sex were tested using linear mixed models using dominant side and sex as fixed factors and patient as random factor. *p* values < 0.05 were considered statistically significant. Statistical analyses were performed using R 3.3.2 (R Foundation, Vienna, Austria) with package ‘cccrm’ [26].

## Results

The mean systematic difference in measured clavicle length between both observers was 0.88 mm (LoA − 2.47 to 4.48). The observers showed an almost perfect agreement [CCC 0.99 (95% CI 0.98–0.99)] and the measuremens of both observers were averaged.

Of the 100 included CT scans, 42 belonged to male and 58 to female patients. The mean age of the patients was 55.5 years (range 18–80 years). 91 patients were right-handed and 9 were left-handed. The clavicle length measurements are presented in Table 1.

**Table 1:** Clavicle length measurements

Clavicle length (mm)	Mean (range)
Side	
Left	147.8 (122.5–175)
Right	146.0 (121.5–171.5)
Gender	
Male	154.8 (130–175)
Female	141.2 (121.5–161)
Dominance	
Dominant	146.0 (121.5–171.5)
Non-dominant	147.9 (122.5–175)

**Fig. 2:**
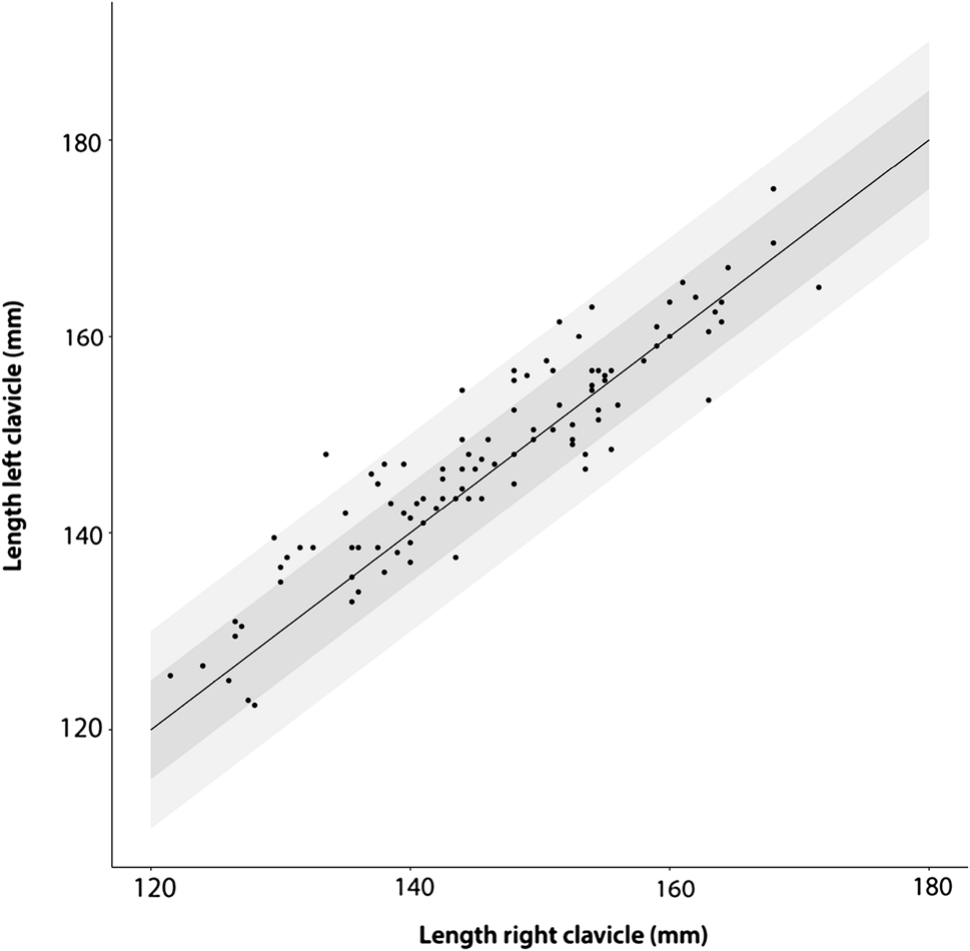
Scatterplot of right versus left clavicle lengths. Dots in the dark grey area represent length differences < 5 mm. Length differences between 5 and 10 mm are within the light grey area

Right clavicles were 1.79 mm (95% CI 0.91–2.66; *p* < 0.001) shorter than the left. The absolute side-to-side length difference (LD) was 3.74 mm (95% CI 3.15–4.32; *p* < 0.001). 28 patients (28%) had an asymmetry between the right and left clavicle of between 5 and 10 mm, and 2% had an asymmetry of more than 10 mm (Fig. 2). Both sex [regression coefficient for males: 13.26 mm (95% CI 9.85–16.67; *p* < 0.001)] and dominant side [regression coefficient for non-dominant side: 1.77 mm (95% CI 0.90–2.63); *p* < 0.001] were associated with clavicle length.

## Discussion

The goal of this study was to assess the side-to-side variation in clavicle length in 100 uninjured, skeletally mature adults using CT scans. To exclude the possibility of variation due to prior clavicular fractures, only patients of whom it was ascertained no prior clavicular fractures had occurred were included. The most important finding in this study is that 30% of the studied population had an asymmetry between the right and left clavicle of 5 mm or more. 2% had an asymmetry of more than 10 mm. This difference could be clinically significant when adhering to the treatment paradigm of surgically treating DMCF when shortened more than 15 mm. There is a large potential for error that could lead over- or under-treatment of the fractured clavicle. It is debatable whether shortening should be used as an indicator for surgery, but, since there is no standardized method of measuring and imaging the fractured clavicle, it also cannot be discarded. A uniform method that takes into account natural asymmetry, patient positioning, imaging technique, and measuring technique could potentially answer this question in the future.

CT scans were used, since this technique provides the most accurate measurements in comparison to others, such as conventional X-rays or clinical measurements [18]. Cunningham et al. [19] were the first to describe an asymmetry of 28.5% of ≥ 5 mm in their researched population. This may lead to the conclusion that quantifying shortening using this method may be unreliable for a significant portion of the population. Unlike Cunningham et al. [19], the present study did investigate the effect of dominant side on clavicular length.

To our knowledge, this study is the first to describe the statistically significant association between clavicle length and dominant side and sex (*p* < 0.001).

A significantly shorter length of the right clavicle and dominant side of, respectively, 1.79 and 1.77 mm was found. The negative association between hand size and dominant side found by Manning et al. [20] seems also to be true for clavicle length and dominance. The number of right-sided dominance found in our study is in concordance with that of the normal population [19].

Some potential limitations have to be discussed. It should be noted that the only way of assessing fractures of the clavicle in the past is using the patient history. This could introduce the chance of recall bias. It can be argued that not everybody who denied having had a fracture of the clavicle would remember the event of clavicle fractures during birth. However, only 2.0–2.7% of deliveries cause a birth-related clavicle fracture, so the influence of this could be deemed insignificant, since an asymmetry ≥ 5 mm in 30% in the studied population was found [27–29]. Another limitation could be that it can be difficult of identify the true extent of the lateral end of the clavicle on CT, particularly on 3D-CT reconstructions. To minimize variability, a training session for the observers and using a standardized measurement methodology was included. A CCC of 0.99 showed this strategy results in a reliable identification of the right point at the lateral end of the clavicle. A third limitation could be the fact that the measurements were performed once by each observer. However, Cunningham et al. [19] reported a strong interobserver reliability with an ICC ranging from 0.70 to 0.86 as well as similar observed length differences (within 1–2 mm) for all observers. Furthermore, a recent study by Goudie et al. [24] used one observer under the assumption CT measurements of the clavicle are precise.

## Conclusion

This study demonstrates that 30% of patients have clavicular side-to-side asymmetry of 5 mm or more. A significant association between clavicle length and dominant side or sex was found (*p* < 0.001). Utilizing a treatment algorithm based upon symmetry, therefore, has a potential for error and can lead over- or under-treatment of the fractured clavicle. To optimize reliability of imaging and measuring shortening of the fractured clavicle, more research is needed. One should consider natural asymmetry, imaging modality and technique, patient positioning, and method for measuring to identify a standardized and reliable method to adequately use the amount of shortening in the treatment algorithm the fractured clavicle.
